# Effectiveness of Oral Nirmatrelvir/Ritonavir vs. Intravenous Three-Day Remdesivir in Preventing Progression to Severe COVID-19: A Single-Center, Prospective, Comparative, Real-Life Study

**DOI:** 10.3390/v15071515

**Published:** 2023-07-07

**Authors:** Dimitrios Basoulis, Aristeidis Tsakanikas, Aikaterini Gkoufa, Aikaterini Bitsani, Georgios Karamanakos, Elpida Mastrogianni, Vasiliki E. Georgakopoulou, Sotiria Makrodimitri, Pantazis-Michail Voutsinas, Panagiota Lamprou, Athanasios Kontos, Stathis Tsiakas, Maria N. Gamaletsou, Smaragdi Marinaki, Nikolaos V. Sipsas

**Affiliations:** 1 Infectious Diseases Unit, Laiko General Hospital, 115 27 Athens, Greece; aristeidis_tsakanikas@hotmail.com (A.T.); katergouf@yahoo.gr (A.G.); kbitsani@gmail.com (A.B.); vaso_georgakopoulou@hotmail.com (V.E.G.); sotiriamakrod@yahoo.gr (S.M.); pantazis88@gmail.com (P.-M.V.); nsipsas@med.uoa.gr (N.V.S.); 2 Department of Pathophysiology, Laiko General Hospital, 115 27 Athens, Greece; medkontos@gmail.com; 3 Haematology Clinic and Bone Marrow Transplantation Unit, Laiko General Hospital, 115 27 Athens, Greece; 4 Emergency Department, Laiko General Hospital, 115 27 Athens, Greece; gkaramanakos@gmail.com (G.K.); elpidamastrogianni@gmail.com (E.M.); magama@med.uoa.gr (M.N.G.); 5 Pulmonology Department, Laiko General Hospital, 115 27 Athens, Greece; lapanagiota@gmail.com; 6 Department of Nephrology and Renal Transplantation, Laiko General Hospital, 115 27 Athens, Greece; stathis.tsiakas@gmail.com (S.T.); smaragdimarinaki@yahoo.com (S.M.); 7 Medical School, National and Kapodistrian University of Athens, 115 27 Athens, Greece

**Keywords:** nirmatrelvir/ritonavir, remdesivir, COVID-19, inverse-probability of treatment weighting, immunocompromised patients

## Abstract

Background: Nirmatrelvir/ritonavir (NMV/r) and three-day course remdesivir (3RDV) have been approved as early treatments for COVID-19 outpatients not requiring supplemental oxygen. Real-life data on the efficacy of antivirals among immunocompromised patients or directly comparing their effectiveness in preventing hospitalization and/or death are scarce. Methods: Prospective, observational study conducted in a tertiary care hospital, from 1 January 2022 until 15 March 2023, during the prevalence of the Omicron variant. Inverse probability of treatment weighting (IPTW) was used to account for differences between treatment groups. Results: We included 521, mainly immunocompromised (56%), patients in our analysis; 356 (68.3%) received 3RDV and 165 (31.7%) NMV/r. Overall, 15/521 (2.9%) patients met the primary end-point of hospitalization at 30 days (3RDV arm: 10/356, 2.8% vs. NMV/r arm: 5/165, 3%, *p* = 1). On IPTW-adjusted univariable analysis, the choice of treatment did not affect outcomes. In multivariable logistic regression analysis, we found that one (OR 0.26, 95%CI 0.07–0.99, *p* = 0.049) or two (OR 0.06, 95%CI 0.01–0.55, *p* = 0.014) vaccine booster shots reduced the risk for adverse outcomes. Conclusion: In our patient population of high-risk, mainly immunocompromised, vaccinated patients during the prevalence of the Omicron variant, NMV/r and 3RDV were equally effective early treatments for the prevention of hospitalization and/or death.

## 1. Introduction

The intensity of the COVID-19 pandemic has waned since the beginning of 2022, in parallel with a significantly reduced risk for disease progression and death due to widespread vaccination, hybrid immunity, and the intrinsic low virulence of the Omicron subvariants. However, COVID-19 remains a serious threat to public health, even in the era of Omicron predominance, as it is associated with significant hospital (15%) and ICU (5%) admissions [1], 5.5% in-hospital mortality [2] and long-term clinical sequelae [3]. It is estimated that approximately 40% of the global adult population is at high risk for progressing to a more serious illness caused by COVID-19 [4]. The use of early therapies (antiviral monoclonal antibodies and small molecules) has been approved for the prevention of disease progression and hospitalizations among high-risk outpatients with comorbidities [5]. Amongst the various preventative measures employed [6,7], two small molecule treatments (nirmatrelvir/ritonavir and remdesivir) have proven effective.

Remdesivir, an approved antiviral treatment for COVID-19, functions as a direct-acting nucleotide prodrug inhibitor, targeting the RNA-dependent RNA polymerase of the SARS-CoV-2 [8]. The PINETREE trial demonstrated that an early three-day course of remdesivir (3RDV) can successfully prevent hospitalization in high-risk patients [9]. Although remdesivir is contraindicated in patients on dialysis because the formulation contains nephrotoxic cyclodextrin, published data on voriconazole, suggest that the brief exposure of three or five days might not lead to toxicity [10,11].

Nirmatrelvir is a small-molecule antiviral that inhibits viral replication by inactivating the SARS-CoV-2 3-chymotrypsin–like cysteine protease enzyme (Mpro) [12] and is co-formulated with ritonavir, a potent inhibitor of CYP3A4, which increases the bioavailability of the active drug [12]. The combination nirmatrelvir/ritonavir (NMV/r) received approval as a treatment to prevent COVID-19 hospitalization in high-risk individuals through the EPIC-HR trial [13]. Due to the inclusion of ritonavir in the regimen, several drug interactions are expected, and physicians are advised to check for these when prescribing the drug. Drug–drug interactions impede the use of NMV/r by patients receiving other medications interfering with the CYP3A4 system. Moreover, NMV/r requires renal dose adjustments [14].

The option of treating individuals as outpatients, with oral or intravenous antimicrobial chemotherapy, thus avoiding unnecessary hospitalizations, is constantly gaining ground worldwide [15]. Early treatment of COVID-19 outpatients with oral antivirals or with short daily infusions is considered a treatment strategy that assisted in turning the tides of the pandemic by reducing hospital admissions and mortality. Considering that monoclonal antibodies have lost effectiveness against currently circulating variants [16], it seems that 3RDV and NMV/r, which maintained their efficacy, remain the only option for early treatment. It should be noted that researchers have identified mutations conferring resistance to both NMV/r and remdesivir in vitro [17,18,19,20], but no clinical failures associated with those mutations have been reported. A remdesivir oral derivative is planned that will confer the same effectiveness combined with ease of use and a better safety profile [21].

The regulatory studies of both NMV/r and remdesivir have been performed before the predominance of the Omicron variant, and mainly among non-vaccinated patients. There are few real-life studies that have attempted to verify the efficacy of early antiviral treatment among vaccinated, high-risk patients with COVID-19, not requiring hospitalization, during the predominance of the Omicron variant. A retrospective study from Israel, among 3761 high-risk patients treated with NMV/r, showed that NMV/r was associated with a greater decrease in the composite end-point of severe COVID-19 and mortality (hazard ratio 0.43, 95% CI 0.85–0.64) [22]. In a study from Singapore, the authors compared the effectiveness of 3RDV vs. the monoclonal antibody sotrovimab (at the time approved and effective for circulating viral variants) and found that even though 17.8% of remdesivir participants proceeded to require supplemental oxygen when studied after propensity score matching, 3RDV would reduce the danger of hospitalization by 82% [23]. Larger studies from the US using propensity score matching showed that NMV/r reduced the risk of deterioration by 85% [24]. Finally, a large population study from Hong Kong compared the effectiveness of NMV/r to molnupiravir and controls. Patients receiving NMV/r had a 40% reduction in a primary composite outcome of disease progression (oxygen supplementation, all-cause mortality, mechanical ventilation, and intensive care unit admission) [25].

Data on the efficacy of early antiviral treatment among immunocompromised outpatients with COVID-19 in the Omicron variant era are scarce. Moreover, only a few studies have attempted a direct comparison between 3RDV and NMV/r, the only available options for early treatment in high-risk patients. In our study, we aimed to investigate the efficacy of 3RDV vs. NMV/r in preventing hospitalization among mainly immunocompromised, vaccinated, high-risk outpatients with COVID-19, during the predominance of the Omicron variant, by using inverse probability of treatment weighting (IPTW) techniques to account for inherent bias when choosing treatments.

## 2. Materials and Methods

### 2.1. Study Design

This is a prospective observational study conducted at the Laiko General Hospital of Athens, a tertiary care, university-affiliated, 580-bed hospital in Athens, Greece. The study period spanned from 1 January 2022 to 15 March 2023 and included all consenting, consecutive patients visiting the hospital’s emergency department with verified PCR SARS-CoV-2 infection and receiving treatment with either NMV/r or 3RDV to prevent hospitalization and/or death. In Greece, the Omicron variant was identified for the first time in December 2021, and from 15 January 2022 onwards, it is the predominant variant [26]. We did not perform PCR genotyping in all study participants, but based on the epidemiology of the pandemic in Greece during the study period, it can be safely assumed that all participants were infected by the Omicron variant [27].

The primary outcome was hospitalization at 30 days. Secondary outcomes included mortality at 30 days and length of hospital stay, hospitalization, and mortality at 90 days.

### 2.2. Study Population

Patients were eligible for inclusion in the study when they had PCR-confirmed COVID-19 infection, without requirements for supplemental oxygen on presentation, but with at least one risk factor for disease progression, as presented in Table 1, regardless of symptoms. Symptomatic patients, however, were excluded from participation if their symptoms had an onset more than five days before the presentation or in the case of either symptomatic or asymptomatic patients if more than three days had passed since the patient’s first positive SARS-CoV-2 PCR results, in accordance with national guidelines [28]. Pregnant women were also excluded from the study. For this study’s purposes, disease onset was defined as symptom onset for symptomatic patients or the date of the first positive test for asymptomatic individuals.

On presentation, the caring physicians collected a complete medical history record, including vaccination information, and performed a thorough physical exam and, depending on the physician’s judgment, laboratory examinations. Immunosuppression was defined by the presence of a primary or acquired immunosuppressive disease or therapy, such as corticosteroids, chemotherapy, immunotherapy, etc. All participants underwent chest X-ray imaging and received arterial blood gas testing. The decision on which early treatment would be given to the patient was made by the caring physician based mainly on relative contraindications and/or drug interactions. All patients were followed-up for 90 days.

### 2.3. Statistical Analysis

Descriptive statistics are presented as counts (%) for categorical variables and as medians (25th and 75th percentile) for non-normally distributed continuous variables or as means ± standard deviation (SD) for normally distributed continuous variables. The normality of distribution was examined using the Kolmogorov–Smirnov test. Group comparisons were performed using the Student’s *t*-test and Mann–Whitney U test for normally and non-normally distributed variables, respectively, and chi-square for categorical variables.

Due to inherent biases in treatment choices, the effect of the treatment group was investigated after performing IPTW to assess the average treatment effect of NMV/r and 3RDV. IPTW assigns weights to each individual case based on a propensity score. In this manner, no individual is excluded from the analysis as would happen with propensity score matching, but a synthetic sample is derived in which the distribution of measured baseline covariates is independent of treatment assignment [29]. The propensity score was calculated using the following variables: age, gender, history of comorbidities (asthma, chronic obstructive pulmonary disease (COPD), hypertension, coronary arterial disease (CAD), congestive heart failure (CHF), chronic kidney disease (CKD), hematological or solid organ malignancy, liver disease, diabetes mellitus, autoimmune disease, immunosuppressive conditions), Charlson comorbidity index (CCI), vaccination status, and time from symptom onset.

Multivariable analyses were performed using binary logistic regression. All variables with a *p*-value < 0.1 in the univariate analysis, as well as variables that would be reasonable to be added to the model based on the known literature, were included. Results of the logistic regression are presented as Odds Ratios (OR), with 95% confidence intervals (CI). A Kaplan–Meier survival curve was employed to demonstrate if there was a difference in the time to hospitalization between the two treatment groups. Statistical findings with a *p*-value < 0.05 were considered statistically significant. The analysis was performed using SPSS Statistics for Windows, 2017 (Version 25.0; IBM Corp., Armonk, NY, USA).

## 3. Results

### 3.1. Participant Characteristics

A total of 521 patients were included in the study analysis with a mean age of 61.8 ± 17.2 years; 57.2% (298/521) were male. Regarding treatment, 356/521 (68.3%) received 3RDV, and 165/521 (31.7%) received NMV/r. Basic demographic and medical history information is presented in Table 2.

The two treatment arms differed significantly, with the 3RDV group being younger (60.2 vs. 65.2 years, *p* = 0.02), suffering proportionately less frequently from COPD or asthma (6.2 vs. 13.3%, *p* = 0.01), hypertension (39.2 vs. 49.7%, *p* = 0.028), and hematological malignancies (15.2 vs. 37%, *p* < 0.001). Patients in the 3RDV group were statistically more likely to have chronic kidney disease (38 vs. 0.6%, *p* < 0.001) and more overall comorbidities, as represented by the CCI scores (*p* = 0.025). There were no differences between the two groups regarding vaccination status or time between disease onset and treatment onset. All patients reported completing the assigned oral treatment, and all patients were recorded receiving all three remdesivir infusions.

### 3.2. Univariable Analysis

Fifteen individuals (2.9%) met the primary end-point, proportionately distributed between the two treatment arms (10/356, 2.8% vs. 5/165, 3%, *p* = 1). Similarly, there were no differences in hospitalization at 90 days (12/356, 3.4% vs. 6/165, 3.6%, *p* = 1) and deaths at 30 days (5/356, 1.4% vs. 1/165, 0.6%, *p* = 0.670), and 90 days (5/356, 1.4% vs. 2/165, 1.2%, *p* = 1). All deaths occurred during hospitalization throughout the 90-day follow-up period. There were also no differences in length of hospital stay (median 11.5 vs. 9 days, *p* = 0.892) between the 3RDV and the NMV/r groups.

**Figure 1:**
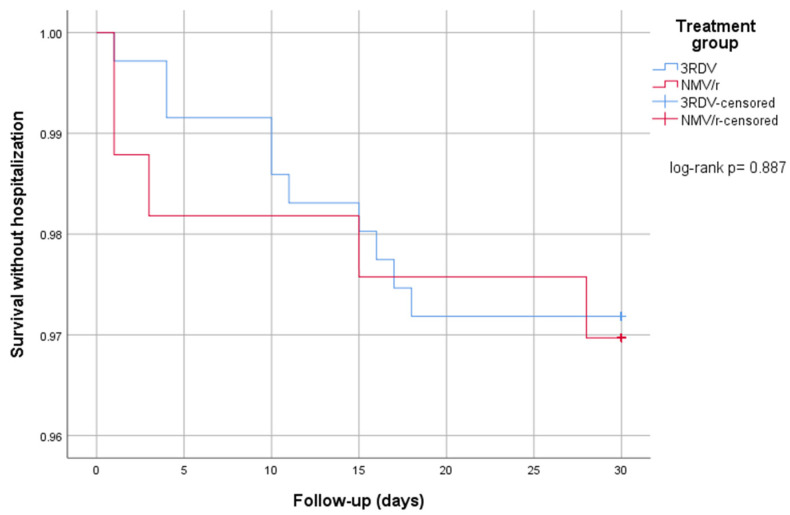
Kaplan–Meier survival analysis for survival without hospitalization at 30 days according to treatment plan. 3RDV: three-day course remdesivir, NMV/r: nirmatrelvir/ritonavir.

On IPTW-adjusted propensity score univariable analysis, there was no difference between the two arms with NMV/r having an OR = 1.4 (95%CI 0.26–7.43, *p* = 0.697) for hospitalization compared to 3RDV (arbitrarily used as reference). Kaplan–Meier survival curves for survival without hospitalization at 30 days are reported in Figure 1. Time from treatment to hospitalization was not different in the two treatment groups (median days 3RDV 13 vs. NMV/r 9, *p* = 0.553).

Univariable analysis in the entire population showed that the only predictor of hospitalization was age (61.5 vs. 70.5, *p* = 0.047) (Table 3). Analysis of the group of only 3RDV recipients could not identify risk factors for hospitalization, whereas, in the NMV/r arm, only CKD, CAD, and vaccination seemed to predict outcomes, but this finding should be taken with caution due to very small numbers (Supplementary Tables S1 and S2).

### 3.3. Multivariable Analysis

The results of the multivariable logistic regression are presented in Table 4. The presence of hypertension maintained a trend to predict adverse outcomes (OR 2.62, 95%CI 0.78–9.2, *p* = 0.083) but ultimately did not have statistical significance.

Patients with three (OR 0.26, 95%CI 0.07–0.99, *p* = 0.049) or four (OR 0.06, 95%CI 0.01–0.55, *p* = 0.014) vaccine doses had lower chances of hospitalization than unvaccinated individuals. Our small numbers made it impossible to discern if such an effect would apply with five vaccine doses.

## 4. Discussion

To our knowledge, our study includes one of the largest real-world published cohorts of COVID-19 patients receiving 3RDV or NMV/r for prevention of hospitalization and/or death. Moreover, it is one of few studies that attempt to compare head-to-head these two treatment options in a cohort of mainly (56%) immunocompromised and vaccinated patients in the era of the Omicron variant prevalence.

We have shown that in our study population of mainly immunocompromised patients, antivirals had an efficacy of 97.1% in preventing hospitalization at 30 days, similar to that found in the pivotal regulatory studies, of 99.25% (13/1736) and 99.3% (2/279) of NMV/r and 3RDV, respectively [9,13]. The slightly reduced efficacy is probably attributed to the large percentage of immunocompromised participants. Although regulatory trials for NMV/r and 3RDV were designed to include high-risk patients, they included only 12 (0.5%) and 23 (4.1%) immunocompromised patients, respectively [3,7]. However, currently, it is precisely this immunocompromised population, and especially the B-cell-depleted patients, that have the highest risk of severe infection [30]. According to Najjar-Debbiny et al. [22], among high-risk outpatients with COVID-19, immunosuppression is associated with a sharp increase in the risk for severe COVID-19 or mortality (hazard ratio 7.36, 95% CI 5.51–9.82). In our real-life study, early antiviral treatment was effective among mainly immunosuppressed high-risk outpatients.

Given the similar efficacy, it would be prudent to focus on aspects of tolerability for these medications. In our study, we had no treatment discontinuations due to side effects for both treatment arms. We did not record minor adverse events, not dictating treatment discontinuation, but regulatory studies showed that both remdesivir and NMV/r seem to be well tolerated, with low rates of treatment discontinuation [31,32].

Our study was conducted during the Omicron era, and all participants were infected with the Omicron variant since in Greece, starting from the end of December 2021, the Omicron variant has completely dominated over the other strains. Of note, a very high proportion of our patients were vaccinated, with 88.5% having received at least one dose of COVID-19 vaccine. This is in contrast with the regulatory trials of both NMV/r and 3RDV, where vaccinated patients had been excluded. We demonstrated additional findings on the antiviral protective effect of vaccination in preventing hospitalizations among participants who had received one or two booster shots. During the Omicron wave, we have seen globally that vaccine effectiveness has waned [33], but we have shown that it is still important for high-risk individuals, especially immunocompromised patients, to be vaccinated. Vaccination has proven effective at reducing the risk of severe outcomes, but also, in a time when global communities are trying to recover from the economic ramifications of the pandemic, it has helped reduce work absenteeism [34].

Hypertension seems to have a deleterious effect on COVID-19 outcomes. Our data demonstrate a trend toward an increase in hospitalization risk among hypertensive patients. This finding is in accordance with several studies that have linked hypertension to increased risk for hospitalization and mortality in COVID-19 patients [35]. The published literature has shown that hypertension treatment with medications that block the renin-angiotensin pathway could reduce the risk of severe disease and ICU admission [36]. Unfortunately, we did not collect relevant information in our cohort to further investigate these associations.

With regards to overall mortality, 38.9% (7/18 at 90 days or 40% for 6/15 at 30 days) of hospitalized patients died. It seems that when 3RDV or NMV/r failed to halt disease progression, it was highly likely that this would turn out to be fatal for the patient. In our study, given the small numbers, it is impossible to determine what factors would be able to predict adverse outcomes.

We have shown that 3RDV and NMV/r had equal efficacy as early treatment for the prevention of severe COVID-19. Recently published trials have also attempted to compare these treatments. Manciulli et al., in a multicenter Italian study using the same technique of IPTW we employed, also attempted to compare sotrovimab, molnupiravir, 3RDV, and NMV/r [37]. In their cohort, sotrovimab (which, since then, has given new circulating variants, has lost its potency) seemed to fare better than the small molecule treatments. Predictors of negative outcomes included malignancies, the presence of multicomorbidity, and delays in treatment initiation. Similar to our results, only 2.3% of patients overall required hospitalization, and 0.3% died. In another Italian study from Pisa, not accounting for treatment choice bias, hospitalizations were more frequent with 3RDV (5.1%) compared to NMV/r (0.8%), although the latter had more adverse events recorded [38]. NMV/r was also faster at reducing viral load as detected by nasal swabs.

One limitation of our study is that we did not include patients who opted out of any available treatments; therefore, there was no control group of untreated, high-risk patients. A comparison with an untreated control group would offer significant information. Mazzitelli et al., in Italy, compared 3RDV to no treatment in a large real-world cohort and found that the use of 3RDV would reduce the risk of hospitalization by 95% and the duration of positivity and symptoms by eight and five days, respectively [39]. Similarly, a recent observational study from Greece compared NMV/r to no treatment and showed that in the first group, only 1.5% required eventual hospitalization, compared to a staggering 55.5% in the control group [32].

Our study has several other limitations. Foremost, the two treatment groups are neither randomized nor similar. We attempted to address this limitation by employing IPTW methods. We used only clinical data and medical history since full laboratory investigations were not available for a large portion of patients included in the study. It is possible that known predictors of adverse outcomes, such as ferritin, lactate dehydrogenase, or neutrophil to lymphocyte ratio, could improve our models [40]. Even though we have data regarding the number of individual vaccine doses, we do not have information with regard to the distance from the last dose. This would be particularly useful when discussing infections during the Omicron waves, where the virus is known to evade the immune system more effectively [41]. We also did not collect data on the tolerability of either treatment or minor adverse events not leading to treatment discontinuation. Finally, our single-center trial might not be applicable to different settings and countries or different periods of the pandemic, as new variants might emerge that make this data obsolete. A prospective randomized cohort study comparing these treatments or a meta-analysis could confirm our real-world findings.

## 5. Conclusions

NMV/r and 3RDV seem to be both equally effective treatments for the prevention of hospitalization and/or death in a population of high-risk, mainly immunocompromised, vaccinated COVID-19 outpatients, infected with the Omicron variant of SARS-CoV-2. Early access to these treatments and being up to date on vaccinations are important, especially for immunocompromised patients, to prevent adverse outcomes.

## Figures and Tables

**Table 1:** Indications for early protective COVID-19 treatments.

**TWO of the Conditions Below**	**Or ONE of the Conditions Below**
Age ≥ 65 years BMI ≥ 35 kg/m^2^ Diabetes mellitus under treatment Chronic kidney disease Chronic liver disease Chronic cardiovascular disease (stroke, coronary artery disease, congestive heart failure, peripheral artery disease, hypertension under treatment) Pulmonary fibrosis Respiratory failure under long-term oxygen therapy Hemoglobinopathies	Age ≥ 75 years Cystic Fibrosis Solid organ malignancy under treatment Hematological malignancy under treatment Primary immunodeficiencies HIV with CD4 < 200 cells/μL Patients under dialysis Transplant recipient or in waiting list for transplantation

BMI: body mass index; HIV: human immunodeficiency virus.

**Table 2:** Baseline characteristics of total population and divided into treatment groups.

	N (%) or Mean ± SD or Median [IQR 25–75]			
	Total (521)	3RDV (356)	NMV/r (165)	*p*
**Age (years)**	61.8 ± 17.2	60.2 ± 17.6	65.2 ± 15.9	0.02
**Male gender**	298 (57.2)	198 (55.6)	100 (60.6)	0.296
**Medical history**				
**COPD/asthma**	44 (8.4)	22 (6.2)	22 (13.3)	0.01
**Hypertension**	221 (42.4)	139 (39.2)	82 (49.7)	0.028
**CAD**	50 (9.6)	38 (10.7)	12 (7.3)	0.264
**CHF**	28 (5.4)	17 (4.8)	11 (6.7)	0.406
**CKD**	136 (26.1)	135 (38)	1 (0.6)	<0.001
**Hematological malignancy**	115 (22.1)	54 (15.2)	61 (37)	<0.001
**Solid organ malignancy**	33 (6.3)	19 (5.4)	14 (8.5)	0.18
**Diabetes mellitus**	124 (23.8)	81 (22.8)	43 (26.1)	0.44
**Liver disease**	16 (3.1)	12 (3.4)	4 (2.4)	0.786
**Autoimmune disease**	50 (9.6)	37 (10.4)	13 (7.9)	0.426
**Immunosuppressed**	292 (56)	215 (60.6)	77 (46.7)	0.003
**CCI**	4 [3–5]	4 [3–6]	4 [2–5]	0.025
**Days till treatment**	1 [0–2]	1 [0–2]	1 [0–2]	0.204
**Vaccination**				0.139
**None**	60 (11.5)	43 (12.1)	17 (10.3)	
**1 dose**	9 (1.7)	8 (2.3)	1 (0.6)	
**2 doses**	49 (9.4)	40 (11.3)	9 (5.5)	
**3 doses**	243 (46.6)	161 (45.5)	82 (49.7)	
**4 doses**	127 (24.4)	84 (23.7)	43 (26.1)	
**5 doses**	31 (6)	18 (5.1)	13 (7.9)	
**Outcomes**				
**30-day hospitalization**	15 (2.9)	10 (2.8)	5 (3)	1
**90-day hospitalization**	18 (3.5)	12 (3.4)	6 (3.6)	1
**Hospital days**	10.5 [4–20]	11.5 [4–26]	9 [4–37]	0.892
**30-day mortality**	6 (1.2)	5 (1.4)	1 (0.6)	0.670
**90-day mortality**	7 (1.3)	5 (1.4)	2 (1.2)	1

SD: standard deviation, IQR: interquartile range, COPD: chronic obstructive pulmonary disease, CAD: coronary artery disease, CHF: congestive heart failure, CKD: chronic kidney disease, CCI: Charlson comorbidity index.

**Table 3:** Univariable analysis depending on the primary outcome of hospitalization.

	N (%) or Mean ± SD or Median [IQR 25–75]		
	No Hospitalization (506)	Hospitalization (15)	*p*
**Age (years)**	61.5 ± 17.3	70.5 ± 13.6	0.047
**Male gender**	286 (56.6)	11 (73.3)	0.290
**Medical history**			
**COPD/asthma**	44 (8.8)	0 (0)	0.387
**Hypertension**	211 (41.8)	10 (66.7)	0.066
**Coronary artery disease**	47 (9.3)	3 (20)	0.167
**Congestive heart failure**	27 (5.4)	1 (6.7)	0.569
**Chronic kidney disease**	132 (26.1)	4 (26.7)	1
**Hematological malignancy**	111 (22)	4 (26.7)	0.752
**Solid organ malignancy**	32 (6.4)	1 (6.7)	0.569
**Diabetes mellitus**	121 (24)	3 (20)	1
**Liver disease**	16 (3.2)	0 (0)	1
**Autoimmune disease**	48 (9.5)	2 (13.3)	0.647
**Immunosuppressed**	282 (55.8)	10 (66.7)	0.443
**Charlson comorbidity index**	4 [2–5]	5 [3–6]	0.079
**Days till treatment**	1 [0–2]	1 [0–2]	0.965
**Vaccination**			0.153
**None**	56 (11.1)	4 (26.7)	
**1 dose**	9 (1.8)	0 (0)	
**2 doses**	46 (9.1)	3 (20)	
**3 doses**	236 (46.8)	7 (46.7)	
**4 doses**	126 (25)	1 (6.7)	
**5 doses**	31 (6.2)	0 (0)	
**Treatment**			1
**3RDV**	345 (68.3)	10 (66.7)	
**NMV/r**	160 (31.7)	5 (33.3)	

SD: standard deviation, IQR: interquartile range, COPD: chronic obstructive pulmonary disease, 3RDV: three-day remdesivir, NMV/r: nirmatrelvir/ritonavir.

**Table 4:** Multivariable logistic regression analysis for the primary outcome of hospitalization.

Variable	OR	95%CI	*p*
**Age per year**	1.03	0.98–1.07	0.213
**Male gender**	2.62	0.78–8.8	0.119
**Hypertension**	2.83	0.87–9.2	0.083
**Charlson comorbidity index per point**	1.08	0.78–1.48	0.649
**Vaccination**			
**None**	Ref	Ref	Ref
**1 dose**	NC	NC	NC
**2 doses**	0.35	0.06–2.12	0.254
**3 doses**	0.26	0.07–0.99	0.049
**4 doses**	0.06	0.01–0.55	0.014
**5 doses**	NC	NC	NC

OR: odds ratio, CI: confidence interval, Ref: reference, NC: not calculable.

## Data Availability

Data can be made available at the Pergamos repository of the National and Kapodistrian University of Athens upon acceptance.

## Supplementary Materials

The following supporting information can be downloaded at: https://www.mdpi.com/article/10.3390/v15071515/s1, Table S1: Univariable analysis in the group of 3RDV-treated patients depending on the primary outcome of hospitalization at 30 days. Table S2: Univariable analysis in the group of NMV/r-treated patients depending on the primary outcome of hospitalization at 30 days.
