# Supplementary material for: Effectiveness of Oral Nirmatrelvir/Ritonavir vs. Intravenous Three-Day Remdesivir in Preventing Progression to Severe COVID-19: A Single-Center, Prospective, Comparative, Real-Life Study
Source: Viruses. 2023 Jul 7;15(7):1515. doi: 10.3390/v15071515 (PMC10383489; doi:10.3390/v15071515)
Supplement: Supplementary file 1 [file viruses-15-01515-s001.zip › viruses-2487837-supplementary.pdf]

Supplementary Table 1: Univariable analysis in the group of 3RDV treated patients depending on the primary outcome of hospitalization at 30 days.

	**N (%) or mean ± SD or median [IQR 25, 75]**		
	**No hospitalization (345)**	**Hospitalization (10)**	**p**
**Age (years)**	59.9 ± 17.6	70.4 ± 13.9	0.061
**Male gender**	190 (55.1)	7 (70)	0.522
**Medical history**			
**COPD/asthma**	22 (6.4)	0 (0)	1
**Hypertension**	133 (38.6)	6 (60)	0.198
**CAD**	37 (10.7)	1 (10)	1
**CHF**	17 (4.9)	0 (0)	1
**CKD**	132 (38.3)	3 (30)	0.748
**Hematological malignancy**	51 (14.8)	3 (30)	0.183
**Solid organ malignancy**	18 (5.2)	1 (10)	0.427
**Diabetes mellitus**	79 (22.9)	2 (20)	1
**Liver disease**	12 (3.5)	0 (0)	1
**Autoimmune disease**	35 (10.1)	2 (20)	0.280
**Immunosuppressed**	207 (60)	8 (80)	0.326
**CCI**	4 [3, 6]	4.5 [4, 7]	0.215
**Days till treatment**	1 [0, 2]	0 [0, 2]	0.993
**Vaccination**			0.270
**None**	40 (11.6)	3 (30)	
**1 dose**	8 (2.3)	0 (0)	
**2 doses**	39 (11.3)	1 (10)	
**3 doses**	155 (45.1)	6 (60)	
**4 doses**	84 (24.4)	0 (0)	
**5 doses**	18 (5.3)	0 (0)	

3RDV: 3-day remdesivir regimen, SD: standard deviation, IQR: Interquartile range, COPD: chronic obstructive pulmonary disease, CAD: coronary artery disease, CHF: congestive heart failure, CKD: chronic kidney disease, CCI: Charlson comorbidity index

Supplementary Table 2: Univariable analysis in the group of NMV/r treated patients depending on the primary outcome of disease hospitalization at 30 days.

	**N (%) or mean ± SD or median [IQR 25, 75]**		
	**No hospitalization (160)**	**Hospitalization (5)**	**p**
**Age (years)**	65 ± 16	70.6 ± 14.5	0.442
**Male gender**	96 (60)	4 (80)	0.649
**Medical history**			
**COPD/asthma**	22 (13.8)	0 (0)	1
**Hypertension**	78 (48.8)	4 (80)	0.210
**CAD**	10 (6.3)	2 (40)	0.043
**CHF**	10 (6.3)	1 (20)	0.295
**CKD**	0 (0)	1 (20)	0.03
**Hematological malignancy**	60 (37.5)	1 (20)	0.652
**Solid organ malignancy**	14 (8.8)	0 (0)	1
**Diabetes mellitus**	42 (26.3)	1 (20)	1
**Liver disease**	4 (2.5)	0 (0)	1
**Autoimmune disease**	13 (8.1)	0 (0)	1
**Immunosuppressed**	75 (46.9)	2 (40)	1
**CCI**	4 [2, 5]	5 [3, 6]	0.111
**Days till treatment**	1 [0, 2]	1 [0, 2]	0.909
**Vaccination**			0.022
**None**	16 (10)	1 (20)	
**1 dose**	1 (0.6)	0 (0)	
**2 doses**	7 (4.4)	2 (40)	
**3 doses**	81 (50.6)	1 (20)	
**4 doses**	42 (26.3)	1 (20)	
**5 doses**	13 (8.1)	0 (0)	

NMV/r: nirmatrelvir/ritonavir regimen, SD: standard deviation, IQR: Interquartile range, COPD: chronic obstructive pulmonary disease, CAD: coronary artery disease, CHF: congestive heart failure, CKD: chronic kidney disease, CCI: Charlson comorbidity index
